# Electrophysiological correlates of semantic pain processing in the affective priming

**DOI:** 10.3389/fpsyg.2023.1201581

**Published:** 2023-09-08

**Authors:** Anna Gilioli, Eleonora Borelli, Luana Serafini, Francesca Pesciarelli

**Affiliations:** ^1^Department of Biomedical, Metabolic and Neural Sciences, University of Modena and Reggio Emilia, Modena, Emilia-Romagna, Italy; ^2^Department of Medical and Surgical Sciences, University of Modena and Reggio Emilia, Modena, Emilia-Romagna, Italy

**Keywords:** affective priming, pain, words, event-related potentials, N400, LPP, semantics

## Abstract

**Introduction:**

Pain plays a fundamental role in the well-being of the individual, and its semantic content may have specific properties compared to other negative domains (i.e., fear and anger) which allows the cognitive system to detect it with priority. Considering the influence of the affective context in which stimuli (targets) are evaluated, it is possible that their valence could be differentially processed if preceded by negative stimuli (primes) associated with pain than negative stimuli not associated with pain. Thus, the present study aims to investigate the electrophysiological correlates of the implicit processing of words with pain content by using an affective priming paradigm.

**Methods:**

Event-related potentials (ERPs) were recorded while participants were presented with positive and negative word targets (not associated with pain) that were preceded by positive, negative (not associated with pain), and pain word primes. Participants were asked to judge the valence of the target word.

**Results:**

Results showed faster reaction times (RTs) in congruent conditions, especially when the negative target was preceded by a pain prime rather than a positive one. ERPs analyses showed no effect of pain at an early-stage processing (N400), but a larger waveform when the pain prime preceded the positive prime on the LPP.

**Discussion:**

These results reaffirm the importance that valence has in establishing the priority with which stimuli are encoded in the environment and highlight the role that pain has in the processing of stimuli, supporting the hypothesis according to which the valence and the semantics of a stimulus interact with each other generating a specific response for each type of emotion.

## 1. Introduction

Given the large number of sensory inputs constantly competing for the individuals’ limited processing resources, the attentional system seems to be able to automatically encode the stimuli present in the environment in terms of priority established based on their affective content, i.e., their evaluation in terms of positive or negative valence (Fazio et al., 1986). For this reason, positive and negative stimuli are processed with priority over neutral stimuli (Johansson et al., 2004; Gross and Schwarzer, 2010), and negative stimuli with priority over positive ones (Wentura, 2000; Rhudy and Williams, 2005), consistently with the importance of detecting negative stimuli for the survival of the individual (*negativity bias*; Ito et al., 1998; Dahl, 2001). This effect has been observed also for stimuli whose valence is not innate but culturally acquired as words (Kanske and Kotz, 2007; Kousta et al., 2009; Yap and Seow, 2014; Goh et al., 2016).

Beyond the affective content of a stimulus, also its semantic content, i.e., the conveyed information or meaning, has a role in modulating the attentional system. Affective and semantic contents refer to different and independent dimensions of stimuli and while the role of the former in modulating cognitive responses is well established, only recently it has been proposed that also the latter may play a part (Witherell et al., 2012; Kveraga et al., 2015; Lindquist et al., 2016; Brooks et al., 2017; Borelli et al., 2018).

Stimuli semantically associated to pain, like words conveying pain or faces expressing pain, usually hold a negative affective content. Yet, a semantic content associated to pain is more salient compared to other negative semantic contents. This is likely because of its relevance for the well-being and survival of the individual (Kveraga et al., 2015; Brooks et al., 2017; Aguado et al., 2018; Yao et al., 2019), which makes the stimulus processing more urgent compared to other negative stimuli with different, less salient, semantic contents. Although pain is usually not considered an emotion, it can be defined as an unpleasant emotional and sensory experience (Raja et al., 2020), which gives it an extremely negative valence (Borelli et al., 2018, 2021). This can be true in the case of real pain as well as potential pain or pain threat. Regardless, pain threat may represent a signal that individuals have to move away to protect themselves and to promote their own survival. By virtue of its evolutionary function, it must be detected by the cognitive system with priority with respect to other stimuli (Yamada and Decety, 2009).

In this context, attention would be biased by a threatening stimulus both in terms of engagement and disengagement (Van Damme et al., 2008a). Attentional engagement and disengagement toward a threatening stimulus refer to the processes by which attention is facilitated in redirecting to and inhibited in withdrawing from the location in which a stimulus perceived as threatening is detected, modulating the response times or accuracy in processing a subsequent stimulus. Distraction, i.e., the shift of attention from the painful stimulus, has often been claimed as an effective strategy to reduce attentional engagement and disengagement; however, a high threatening value of the pain stimulus appears to be a powerful enough feature to disrupt such efficacy by reducing engagement in the distraction task (Van Damme et al., 2008b). These attentional effects are modulated by a number of variables, like task difficulty, as per the attentional-capacity models, or the individual relevance attributed to the stimulus, as per the attention-bias models (Vuong et al., 2018).

However, pain also holds a pro-social value when it implies an approach response toward someone else in pain (Yamada and Decety, 2009). A review by Betti and Aglioti (2016) reported numerous studies in agreement that observing individuals expressing or experiencing pain activates the same brain areas involved in the physical experience of pain itself, generating a sensory, perceptive, and behavioral simulation as a first-hand pain experience (Borelli et al., 2018).

Not only the attention but also the behavioral response to the stimulus seems to be guided by valence (Mouras and Lelard, 2021). Based on the motivational priming theory (Lang, 1995; Davidson and Irwin, 1999; LeDoux, 2000), emotions prime two motivational systems which guide behaviors: the aversive system, which facilitates avoidance/withdrawal responses towards negative stimuli, and the appetitive system, which promotes approaching responses towards positive stimuli (Lang et al., 2000, 2005; Bradley et al., 2001; Lang and Bradley, 2007; Horslen and Carpenter, 2011). The activation of these two motivational systems seems to produce subjective responses to emotions; on the contrary, their impairment may result in emotional deficits. On one hand, valence defines which of the two systems activates; on the other hand, the arousal seems to determine the intensity of activation (Rhudy and Williams, 2005). In this regard, the affective context in which a stimulus is embedded plays a critical role in its processing. When the aversive system is pre-activated by negative emotional stimuli present in the environment, it can indeed be facilitated in the generation of avoidance behaviors; on the contrary, it can be inhibited if pre-activated by positive ones (Meagher et al., 2001).

In literature, an experimental paradigm massively used to investigate how the affective context affects the evaluation of the stimulus in terms of positivity or negativity is the affective priming (Gibbons et al., 2018; Hu and Liu, 2019). Affective priming refers to the influence of emotionally charged stimuli on subsequent evaluations or reactions. According to this paradigm, the elaboration of a first stimulus (prime) may facilitate or inhibit the subsequent behavioral response to a second stimulus (target) if the two stimuli are congruent or incongruent, respectively, in terms of valence (e.g., prime HOLIDAY - target TRIUMPH vs. prime JOYFUL - target STINK).

In agreement with the spreading activation theory (Fazio et al., 1986; Murphy and Zajonc, 1993), the priming effect is due to the fact that the valence of the prime pre-activates the network of concepts associated with it, facilitating the subsequent processing of the target if its affective meaning is represented in this network of concepts, through a mechanism similar to one underlying the semantic priming (Neely, 1991). This agrees with the motivational priming theory: when the aversive system is pre-activated by negative stimuli, the individual will be facilitated in implementing avoidance behaviors. On the contrary, this avoidance response will be inhibited when the appetitive system is pre-activated by positive stimuli (an incongruent condition between prime and target).

Although the affective congruency effect, named priming effect, seems to be consistent for positive valence stimuli (Aguado et al., 2013; Contreras-Huerta et al., 2013; Gibbons et al., 2014), results are not much coherent as regarding negative stimuli. It is not clear whether the negative valence information facilitates (Paulmann and Pell, 2010; Meng et al., 2013) or inhibits (Song et al., 2019; Wu et al., 2021) the processing of subsequent negative stimuli.

Because attention determines what information we focus on and process in the environment by allocating cognitive resources, it might play a significant role in influencing the magnitude of the affective priming effect, modulating the extent to which primes and targets are processed (Seib-Pfeifer et al., 2020). Prime valence is processed automatically under defined conditions, for example when it has a highly motivational relevance for the individual (Codispoti et al., 2007), or when the evaluative dimension is goal-relevant (Spruyt et al., 2018; Rohr and Wentura, 2022). Regardless, it is likely that an increased affective prime processing will boost the priming effect. Conversely, a decreased affective prime processing will likely dampen the priming effect, decreasing the chances of an affective misattribution (Spruyt et al., 2018). Similarly, an increased target processing will likely result in a target evaluation based on its properties rather than solely on the valence of the preceding prime, reducing the priming effect, while a decreased target processing will likely result in a target evaluation more affected by the valence of the preceding primes, minimizing the priming effect (Spruyt et al., 2018). Attention to the target may be reduced in the case of a relevant affective and semantic meaning of the prime.

Even the neural dynamics of this interaction are not clearly understood (Herring et al., 2011; Eder et al., 2012; Comesaña et al., 2013; Hietanen and Astikainen, 2013; Diéguez-Risco et al., 2015; Hartigan and Richards, 2016; Aguado et al., 2018; Song et al., 2019). Event-related potentials (ERPs) indeed represent an online measure of such an effect with a temporal resolution within millisecond range. In literature, several studies on affective priming showed that the incongruency of valence between two stimuli can be indexed by ERPs components as the N400 (Zhang et al., 2006, 2010; Steinbeis and Koelsch, 2011; Eder et al., 2012; Hietanen and Astikainen, 2013), which is a negative-ongoing wave peaking around 400 ms after stimulus onset typically associated with the violation of semantic content (Kutas and Hillyard, 1984). Despite most of the studies found this effect, some reported a null effect (Herring et al., 2011), or even a reverse priming effect with a greater amplitude of the N400 in affective congruent conditions (Paulmann and Pell, 2010; Aguado et al., 2013; Wang and Zhang, 2016). In addition, another ERP component often modulated by the affective incongruency is the late positive potential (LPP) can appear in a window between 400 and 700 ms after stimulus onset and is sensitive to the evaluation of properties of stimuli and to the inconsistency of valence (Herring et al., 2011; Aguado et al., 2013; Comesaña et al., 2013; Hietanen and Astikainen, 2013; Hartigan and Richards, 2016). As for the N400, results are still inconsistent with some studies reporting no effect (Wu et al., 2021) or even a reverse priming effect (Eder et al., 2012; Hartigan and Richards, 2016) due to several overlapping components that appear in that time window. In particular, few studies pointed out how an earlier phase of the LPP (400–600 ms) may indicate the automatic allocation of attention to salient stimuli, while a later phase (post 600 ms) is affected by the top-down influence explicitly interpret the stimulus (Olofsson et al., 2008) or by contextual factors (Foti and Hajcak, 2008). The inconsistency of these results may lie in the fact that the negative valence is generally treated as a single semantic domain when, on the contrary, it embraces a heterogenous group of semantic categories (Rossell and Nobre, 2004). For this reason, it is possible to speculate that when the cognitive system needs to determine the priority of a stimulus, the negative valence of the stimuli interacts with their semantic content generating specific responses for each type of emotion (Fazio et al., 1986). Thus, pain may represent an appropriate model to understand if the specificity of the semantic content of a stimulus present in the environment can interact with the valence to the point of influencing the subsequent elaboration of positive and negative information.

So far, only a few studies demonstrated that the semantics of pain embedded in pictures (Meng et al., 2013; Cameron et al., 2017), faces (Burton et al., 2005; Chiesa et al., 2015, 2017), and words (Yamada and Decety, 2009; Grynberg and Maurage, 2014; Richter et al., 2014; Swannell et al., 2016; Meconi et al., 2018) can be processed in an automatic and early way to the point of influencing the response to subsequent pain stimuli. According to the motivational priming theory, the negative emotional information contained in a stimulus can pre-activate the aversive system and enhance both the physiological and behavioral response to a following pain stimulus (Yamada and Decety, 2009; Meng et al., 2013; Grynberg and Maurage, 2014; Richter et al., 2014; Swannell et al., 2016; Cameron et al., 2017; Meconi et al., 2018). This means that the individual previously exposed to negative information will be more likely to rapidly respond to a pain stimulus by its aversive nature (Yamada and Decety, 2009). In addition, a study recently conducted in our laboratory (Gilioli et al., 2023), increasingly corroborated these results showing that the semantics of pain content in the prime can also facilitate the processing of a negative target not associated with pain. Nevertheless, there is little evidence that showed a reverse effect reporting that the processing of a negative prime might also inhibit the subsequent elaboration of a pain stimulus (Burton et al., 2005; Song et al., 2019). Almost no studies have investigated the neural dynamics of this effect on pain and outcomes are still unclear (Sessa et al., 2014; Swannell et al., 2016; Meconi et al., 2018; Song et al., 2019).

In light of this, the present research aimed at investigating the time course of the implicit processing of pain words, analyzing the ERPs correlates of this effect. For this purpose, we replicated our previous behavioral experiments (Gilioli et al., 2023) using the EEG technique. In Gilioli et al. (2023), we adopted an affective priming paradigm and presented healthy participants with prime words with positive and negative valence (associated and not-associated with pain) and target words with positive and negative valence (not-associated with pain). Participants had to evaluate the valence of the target (valence judgment task) by pressing one of two buttons.

In the present study, we recruited a different sample of participants and asked them to perform the same task on the same stimuli while recording their electrophysiological activity as well as their reaction times (RTs).

Our first goal was to confirm the behavioral findings of our prior work, i.e., a priming effect for pairs of negative words only if the prime had pain-related semantics, as shown by faster reaction times and better accuracy scores. This would support the idea that the processing of a stimulus semantically associated to pain can possibly enable the individual to respond more quickly to upcoming negative information in the environment by allocating the appropriate number of resources for generating a response.

Our second goal was to study the electrophysiological correlates of this effect by focusing on two main ERPs components, the N400 and LPP. To the best of our knowledge, this is the first study to apply ERP component analysis to an affective priming paradigm involving word stimuli associated with pain. We hypothesized that if pain-related semantics elicit a distinct response, then these components would capture it to a greater extent at an electrophysiological level. However, due to the limited and inconclusive nature of previous research, it remains unclear whether this effect is present and in what direction. Therefore, it is reasonable to consider the possibility that the priming effect may also occur in other ERP time windows.

## 2. Methods

### 2.1. Ethics statement

This study was carried out in accordance with the recommendations of the “Italian Association of Psychology” (AIP) Ethical Guidelines (Codice Etico),^1^ and was approved by the local Ethical Committee of the University of Modena and Reggio Emilia. All subjects gave written informed consent in accordance with the Declaration of Helsinki.

### 2.2. Participants

Thirty-seven students at the University of Modena and Reggio Emilia, all females (age range: 19–51 years.; mean age = 25.16; SD = 7.49) participated in the experiment for course credit. All participants were right-handed (L.Q. = 91.7) as assessed by the Italian version of the Edinburgh Handedness Inventory (Oldfield, 1971), and they had normal or corrected-to-normal visual acuity, no history of neurological or mental disorders, and they were Italian native speakers.

Three participants were excluded from the analyses: the first due to a recording error of the experimenter, the second started to perform the task before the recording was initiated, and the last needed to be excluded since the experiment was interrupted by an external issue. Therefore, the statistical analyses were performed on 34 female subjects (age range:19–51 years; mean age = 24.65; SD = 7.21). The sample size was established based on heuristic evaluations of the literature on affective priming, which reports numerous studies with samples of 22–33 subjects (Yamada and Decety, 2009; Wu et al., 2021). We also conducted *a posteriori* sensitivity power analysis (Lakens, 2022) using G*Power 3.1 (Faul et al., 2007) according to which given *N* = 34, α = 0.05 and a power = 80% a minimum partial equal to η^2^ = 0.1968 was found, consistent with the literature on this topic.

The choice of selecting an entire sample of female participants was based on our previous study (Gilioli et al., 2023) in which we found gender differences in the priming effect: indeed, females reported a significant priming effect when asked to evaluate a positive target preceded by a positive prime rather than a pain prime. This effect was not found in males.

### 2.3. Stimuli

Overall, 256 Italian words (both adjectives and nouns) were adopted for this study, among which 32 negative words associated with pain (henceforth, pain words; e.g., *ferita*, injury), 96 negative words not associated to pain (henceforth, negative words; e.g., *vandalo*, vandal), and 128 positive words (e.g., *vita*, life; for the complete word list and English translation, see Supplementary Table S1). Positive and negative words were selected from the Italian version of the ANEW database (Affective Norms for English Words; Montefinese et al., 2014), while pain words were selected from the WOP database (Words of Pain, WOP; Borelli et al., 2018). Pain words were chosen based on their pain-relatedness scores (Borelli et al., 2018), which had to be in the range between 6 and 7 on a rating scale from 1 (not at all associated with pain) to 7 (extremely associated with pain). The three categories of words were controlled for the main psycholinguistic and affective variables that are known to affect the time it takes to process a word, namely familiarity, length in letters, valence, and arousal (see Table 1 for descriptive statistics). Each prime-target pair was also controlled for semantic relatedness (Pedersen et al., 2004).

**Table 1:** Descriptive statistics of familiarity, length in letters, valence, and arousal for the three word categories (i.e., positive, negative, and pain stimuli).

		Familiarity M (SD)	Valence M (SD)	Arousal M (SD)	Length M (SD)
Prime	Positive words	5.05 (±0.46)	5.99 (±0.32)	4.85 (±0.63)	7.44 (±2.11)
	Negative words	4.71 (±0.74)	1.83 (±0.24)	5.06 (±0.50)	8.34 (±1.7)
	Pain words	4.71 (±1.13)	1.67 (±0.39)	5.25 (±0.7)	8.44 (±2.37)
Target	Positive words	5.05 (±0.58)	5.97 (±0.30)	5.06 (±0.45)	7.47 (±1.97)
	Negative words	4.76 (± 0.6)	1.85 (±0.23)	4.82 (±0.60)	7.77 (±1.72)

**Figure 1:**
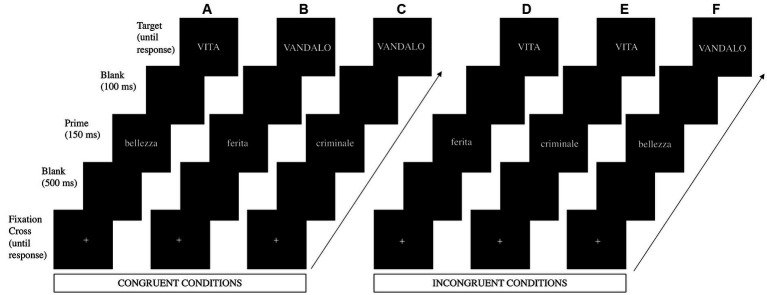
Time sequence of events during an illustrative **(A)** positive prime – positive target trial, **(B)** pain prime – negative target trial, **(C)** negative prime – negative target trial, **(D)** pain prime – positive target trial, **(E)** negative prime – positive target trial, and **(F)** positive prime – negative target trial. The illustrative trials a, b, and c represent the congruent condition, and the illustrative trials d, e, and f represent the incongruent condition. Briefly, a fixation cross (+) was displayed on the screen. Once participants pressed the start button, they were presented in this order with a blank for 500 ms, the prime stimulus for 150 ms, another blank for 100 ms, and the target stimulus, which remained on the screen until the participants’ response. Participants were instructed to evaluate the valence of the target stimulus and to press a button if positive or another button if negative. Adapted with permission from Gilioli et al. (2023).

The 256 words were divided into 128 prime stimuli and 128 target stimuli. Prime stimuli included 32 negative words/prime, 32 pain words/prime, and 64 positive words/prime, whereas target stimuli comprised the remaining 64 negative words/target and the remaining 64 positive words/target. Both prime words and target words were presented 4 times during the entire experiment in 4 blocks and paired to form 512 prime - target pairs (Figure 1). Thereby, we obtained 256 congruent pairs (128 positive prime - positive target; 64 pain prime – negative target; 64 negative prime – negative target), and 256 incongruent pairs (128 positive prime - negative target; 64 negative prime - positive target; 64 pain prime - positive target).

Participants performed four blocks. Within each block, a list of 128 out of 512 prime-target pairs was presented each in a separate trial in a randomized order. The lists were randomized among participants.

### 2.4. Procedure

The experiment was implemented in E-prime software (Version 3; Psychology Software Tools, Pittsburgh, PA) and was presented as a study on how people categorize stimuli based on their positive or negative valence. All stimuli were presented in the center of a 17 CRT monitor synchronous with the screen refresh [Philips 107B; refresh rate = 60 Hz (16.67 ms)] that was positioned at eye level approximately 70 cm in front of the participant, such that each stimulus subtended 1.2–4.1° of horizontal visual angle and 0.5° of vertical visual angle. As shown in Figure 1, each trial began with a fixation cross (+) presented in the middle of the screen and stayed there until participants pressed a button to start the trial. Then a black screen (blank) was displayed for 500 ms and replaced by a prime stimulus lasting 150 ms, followed by another blank lasting 100 ms. Once the blank disappeared, a target stimulus appeared and remained on the screen until the participant’s response. The primes and the targets were presented in the center of the screen in white lowercase letters for the former and in uppercase letters for the latter (20-point Calibri bold font) on a black background. The interstimulus interval was set up at 1000 ms after the participant’s response. Participants were instructed to evaluate, as quickly and accurately as possible, whether the target was a positive or negative word (valence judgment task) and to respond by pressing one of two buttons, which were counterbalanced (left and right) across participants. Participants performed a practice session consisting of 16 trials (half congruent pairs and half incongruent pairs) prior to study onset to ensure that they understood the task. Stimuli in the practice session were different from the experimental ones. The experiment lasted approximately 35 min.

### 2.5. Questionnaires

To assess for individual differences in pain processing, we administered three questionnaires at the end of each experimental procedure: the Italian version of Behavioral Approach/Inhibition System Scale (BIS/BAS scale; Leone et al., 2002) which evaluates the activation and inhibition system; the Italian version of Pain Catastrophizing Scale (PCS-I, Monticone et al., 2012) which measures the individual disposition in pain anxiety and catastrophizing; and the Italian version of the Interpersonal Reactivity Index (IRI, Albiero et al., 2006) which is a measure the dispositional response empathy by integrating affective and cognitive components (e.g., perspective taking, personal distress).

### 2.6. EEG recording and analysis

The EEG data is recorded continuously *via* 64 active electrodes (ActiCap Slim, BrainProducts) placed on the scalp according to the International 10–10. Electrical activity was amplified and sampled at 1000 Hz by a 24-bit ActiCHamp Plus System (BrainProducts) and recorded with BrainVision recorder software (BrainProducts, version 1.25.0101) running on a Windows 10 computer. All electrodes were recorded with the online reference located at FCz. Two electrodes were placed over the left and right mastoids to serve as an offline reference, two were placed at the external ocular canthi of both eyes to monitor horizontal eye movements (HEOG) and one was placed under the left eye to monitor blinks (VEOG). Electrical impedances were kept below 20 kΩ.

Brain Vision Analyzer 2 (Brain Products, Gilching, Germany) was used to perform off-line signal processing analyses. The EEG signal was bandpass filtered between 0.1 and 80 Hz and referenced offline to the average activity of the two mastoids. Artifact activity was rejected using a semiautomated procedure, with artifacts identified by the following criteria: Gradient, with 75 μV maximal allowed voltage step; Max–Min with 200 ms maximal allowed absolute difference; Low activity, with 0.5 μV/100 ms lowest allowed activity. Data with excessive blinks were adaptively corrected using ICA. 1,000-ms epochs containing the ERP elicited by the target word were extracted. A 200 ms pre-stimulus baseline was used in all analyses. Segments including artifacts due to activity exceeding ±100 μV in amplitude were rejected.

The data has been filtered at 30 Hz with the sole purpose of better graphic visualization. The statistical analyses were conducted on the data initially filtered at 0.1–80 Hz. Based on visual inspection of grand average ERP waveforms and in line with previous literature, the following components were identified for target onset at frontal (F3, Fz, F4), central (C3, Cz, C4), and parietal (P3, Pz, P4) scalp sites: N400 from 300 to 500 ms after target onset; LPP from 500 to 700 ms after target onset. For each ERP component amplitude was measured as mean activity within the respective time window.

### 2.7. Statistical analysis

Statistical analyses were performed using JASP software (JASP Team, 2022 Version 0.16.3).

The analysis of accuracy scores was initially performed. RTs and ERP analyses were then carried out on trials with correct responses. Individual RTs exceeding ±2 standard deviations (SD) were excluded from the analysis.

To control for potential confounding effects of primes and targets familiarity, length in letters, valence, arousal, and semantic relatedness we added them as covariates in four analyses of covariance on stimuli RTs and accuracy, one with prime valence and one with prime semantics as a factor.

At the behavioral level, to investigate the role of the prime valence, we performed repeated-measures 2 × 2 ANOVAs on the accuracy rates and the mean RTs with prime valence and target valence as within-subject factors. To investigate the role of the semantic content of prime, we performed repeated-measures 3 × 2 ANOVAs on the accuracy rates and the mean RTs with prime semantics and target valence as within-subject factors. To examine significant interactions, we performed planned paired samples t-tests based on *a-priori* hypotheses. In the 3 × 2 ANOVAs on prime semantics, 64 positive words were compared to negative words, of which 32 were unrelated to pain and 32 were related to pain. For this reason, this analysis resulted in not having the same power as the 2 × 2 ANOVA on prime valence and some effects detected in the latter might appear to weaken in the former. For this reason, both ANOVAs are meaningful to the aim of the study.

At the ERP level, ERP effects time-locked to the onset of the target were evaluated considering 6 clusters of electrodes representing the mean amplitude of three electrodes in close position: Anterior (F3, Fz, F4), Central (C3, Cz, C4), Posterior (P3, Pz, P4), Left (F3, C3, P3), Midline (Fz, Cz, Pz), Right (F4, C4, P4).

A repeated-measures 2×2x3x3 ANOVA was conducted on mean ERP amplitudes with prime valence (positive, negative), target valence (positive, negative), longitude (anterior, central, posterior), and latitude (left, midline, right) as within-subject factors. Secondly, to consider the effect of the semantic content associated to the negative prime, a repeated-measures 3x2x3x3 ANOVA was performed on ERP amplitudes with prime semantics (positive, negative, pain-related), target valence (positive, negative), longitude (anterior, central, posterior), and latitude (left, midline, right) as within-subject factors. To further understand the nature of the interactions, both analyses were followed by separate ANOVAs which were run on the positive and negative target valence. Additionally, *post-hoc* mean comparisons were employed to further examine significant interactions.

In addition, we analyzed the influence of individual differences in pain processing measured by the above-mentioned questionnaires on the behavioral and ERP effects. For each subscale of the questionnaires, the correlation with accuracy scores and RTs Δ congruent-incongruent conditions was measured by the Spearman coefficient for non-parametric measures. As well the correlation with ERP amplitudes of all electrodes Δ congruent-incongruent conditions was measured by the Spearman coefficient for non-parametric measures. This was calculated for both time windows (300–500 and 500–700 ms).

To account for violations of sphericity, the Greenhouse–Geisser procedure was used to correct degrees of freedom: only corrected significance levels are reported. The level of significance for all statistical analyses was set to *p* < 0.05. Holm correction was applied for multiple comparisons and only corrected *p*-values are reported. The main effects of prime valence and target valence in the omnibus ANOVAs were not central to the questions under study. Therefore, they are reported but not discussed. Here, we discussed only the interaction between prime valence and target valence which was of interest to the study. In the separate ANOVAs for the target valence, the main effect of the prime valence was crucial to the analyses: for this reason, it has been discussed.

## 3. Results

### 3.1. Behavioral results

Results from the two ANCOVAs on stimuli accuracy did not reveal any confounding effect of prime and target familiarity, length, valence, arousal, semantic relatedness neither with prime valence nor with prime semantics as a factor. Results from the two ANCOVAs on stimuli RTs did reveal a possible confounding effect of target length (*p* < 0.001) when prime valence was a factor and a possible confounding effect of target length (*p* < 0.001) and target arousal (*p* = 0.008) when prime semantics was a factor. Because the overall results with and without these potentially confounding variables were the same, they have not been included in the analyses on participants’ RTs and accuracy and will not be further discussed.

Overall, 6.6% of trials were excluded from the analyses because the RTs exceeded ±2 SD.

In order to investigate the role of the prime valence, we performed repeated-measures 2×2 ANOVAs on the accuracy rates and the mean RTs. The analysis on the accuracy scores showed a significant main effect of target valence [*F*(1,33) = 6.13, *p* = 0.019, η_p_^2^ = 0.16] so that responses to the negative target (*μ* = 0.96, *SE* = 0.0008) were more accurate than to the positive one (*μ* = 0.94, *SE* = 0.009).

**Figure 2:**
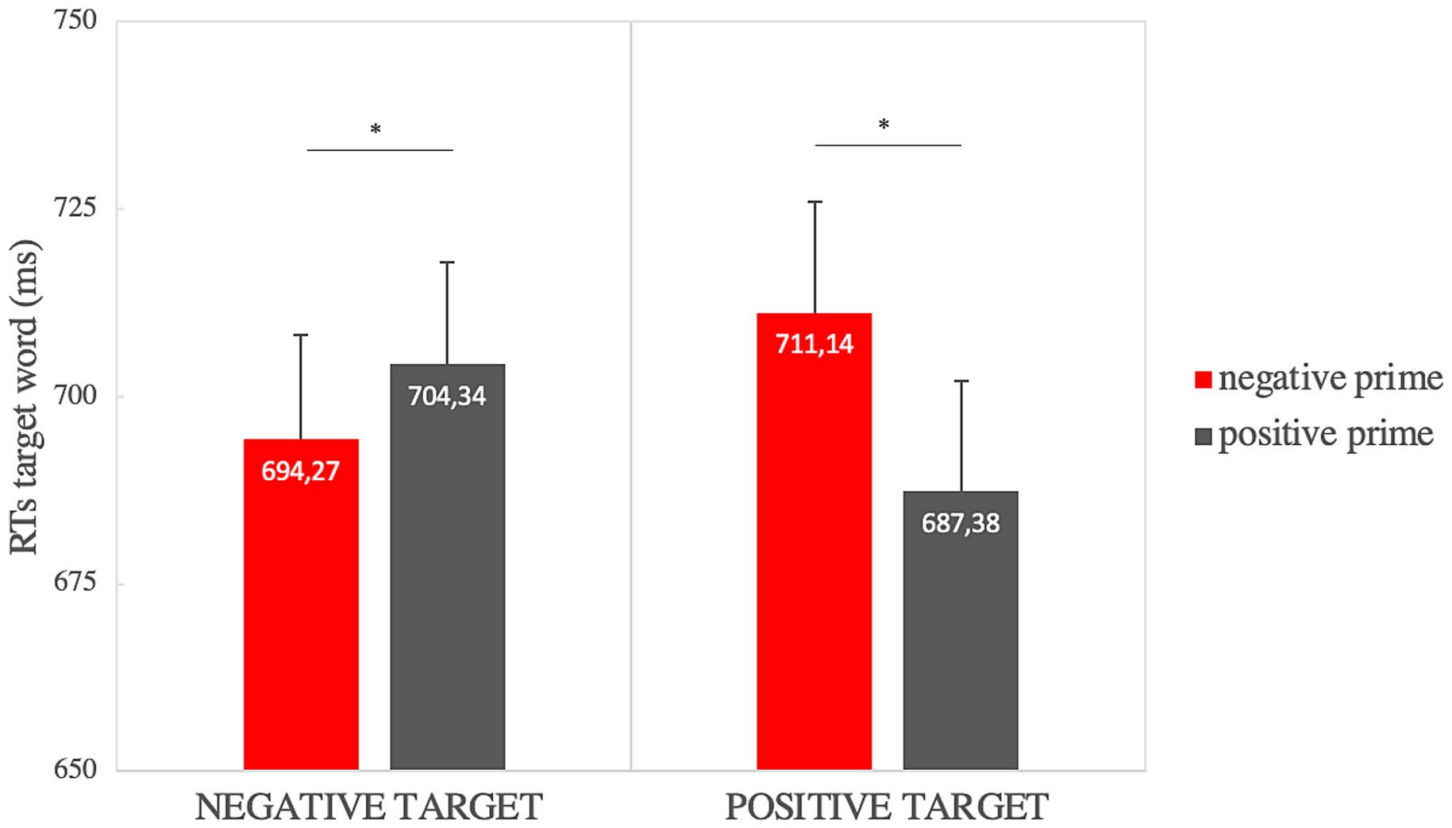
Affective priming effect on RTs for the “valence” factor (in the graph significant comparisons are indicated with *: this highlights the priming effect for positive and negative targets). Error bars represent standard errors of the mean.

The analysis on RTs showed a significant main effect of prime valence [*F*(1,33) = 8.56, *p* = 0.006, η_p_^2^ = 0.21] so that the positive prime (*μ* = 695.86, *SE* = 8.48) was elaborated faster than the negative one (*μ* = 702.71, *SE* = 8,43), and a significant prime valence × target valence interaction [*F*(1,33) = 12.289, *p* = 0.001, η_p_^2^ = 0.271]. Paired sample t-tests showed significantly faster RTs when the positive target was preceded by a positive prime (*μ* = 687.38, *SE* = 14.74) rather than a negative prime (*μ* = 711.14, *SE* = 14.85) [*t*(33) = −4.4, *p* = 0.002 *p* ≤ 0.001, Cohen’s *d* = −0.75]; and significant faster RTs when the negative target (*μ* = 694.27, *SE* = 13.94) was preceded by a negative prime rather than a positive one (*μ* = 704.34, *SE* = 13.57) [*t*(33) = −1.89, *p* = 0.034, Cohen’s *d* = −0.32], as shown in Figure 2.

**Figure 3:**
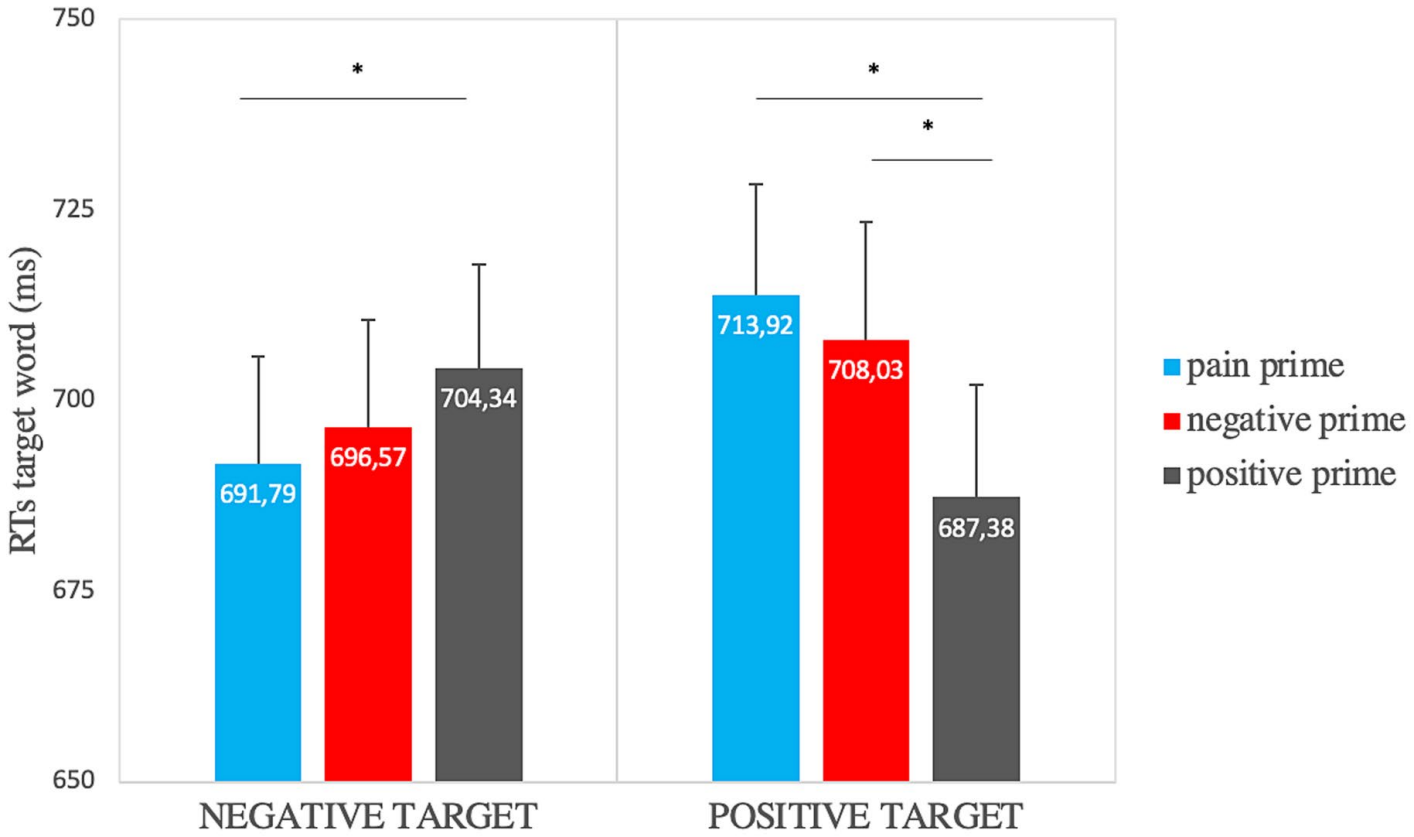
Affective priming effect on RTs for the “semantic” factor (in the graph significant comparisons are indicated with *: this highlights the priming effect for positive and negative targets). Error bars represent standard errors of the mean.

To investigate the role of the prime semantics, we performed repeated-measures 3×2 ANOVAs on the accuracy rates and the mean RTs. The analysis on accuracy rates showed a significant main effect of target valence [*F*(1,33) = 5.58, *p* = 0.024, η_p_^2^ = 0.15] so that responses to the negative target (*μ* = 0.96, *SE* = 0.001) were more accurate than to the positive one (*μ* = 0.94, *SE* = 0.007). The analysis on RTs showed a significant main effect of prime semantics [*F*(1.8, 60.9) = 3.15, *p* = 0.05, η_p_^2^ = 0.09] so that the positive prime (*μ* = 695.86, *SE* = 8.48) was elaborated faster than the negative (*μ* = 702.3, *SE* = 5.73) and the pain one (*μ* = 702.86, *SE* = 11.06); and a significant prime semantics x target valence interaction [*F*(1.5, 49.85) = 10.35, *p* ≤ 0.001, η_p_^2^ = 0.24]. Paired samples t-tests showed significantly faster RTs when the positive target was preceded by a positive prime (*μ* = 687.38, *SE* = 14.74) rather than a pain prime (*μ* = 713.92, *SE* = 14.49) [t (33) = −4.4, *p* = 0.003, Cohen’s *d* = −0.75] or a negative prime (*μ* = 708.03, *SE* = 15.50) [t (33) = −3.59, *p* = 0.003, Cohen’s *d* = −0.62] and significantly faster RTs when the negative target was preceded by a pain prime (*μ* = 691.79, *SE* = 14.07) rather than a positive one (*μ* = 704.34, *SE* = 13.57) [t (33) = −2.21, *p* = 0.017, Cohen’s *d* = −0.38] (Figure 3).

### 3.2. ERP results

**Figure 4:**
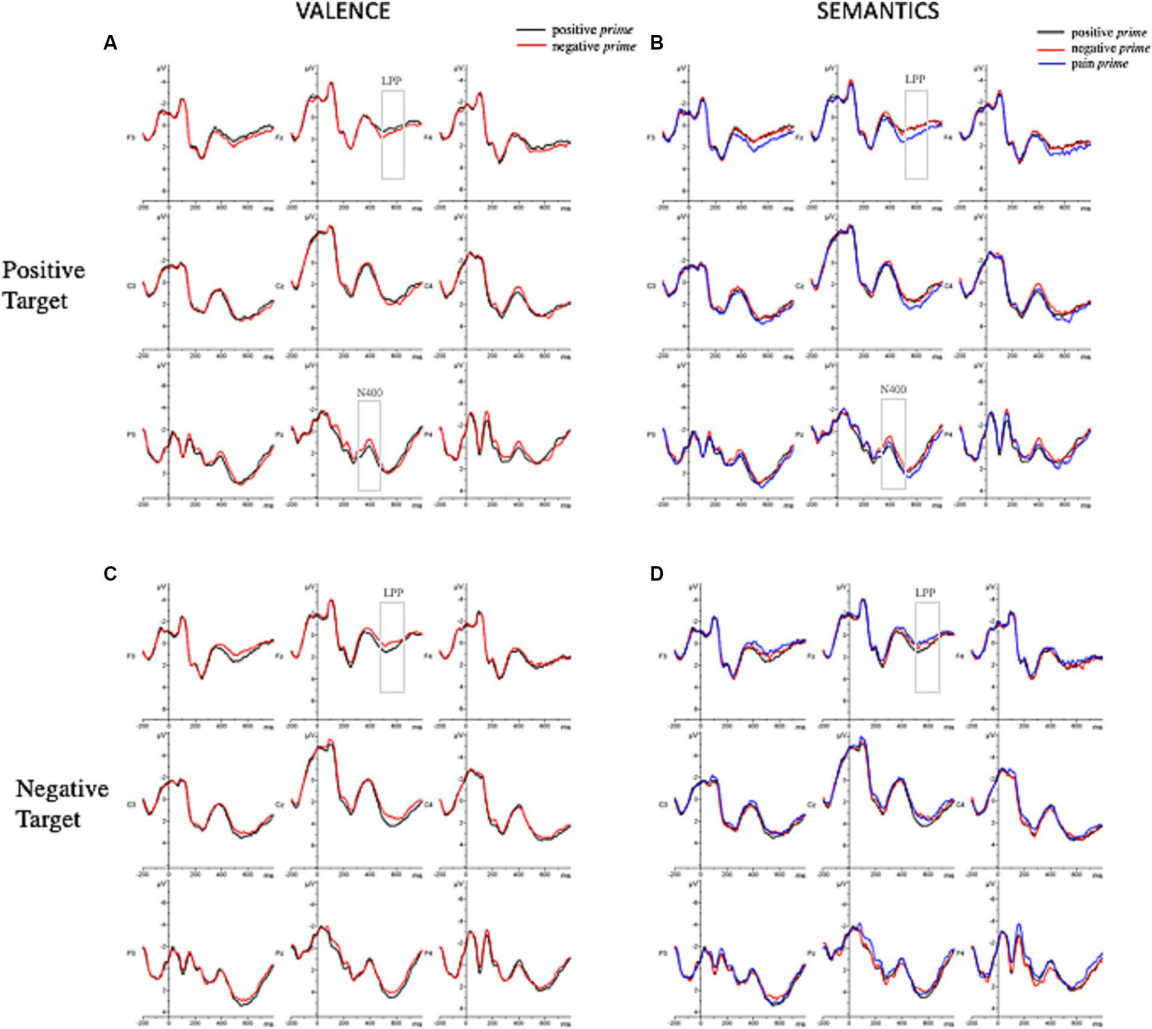
Grand-averaged ERP waveforms elicited by positive and negative target words for the valence manipulation condition **(A,C)** and the semantic manipulation condition **(B,D)** as a function of prime type.

**Figure 5:**
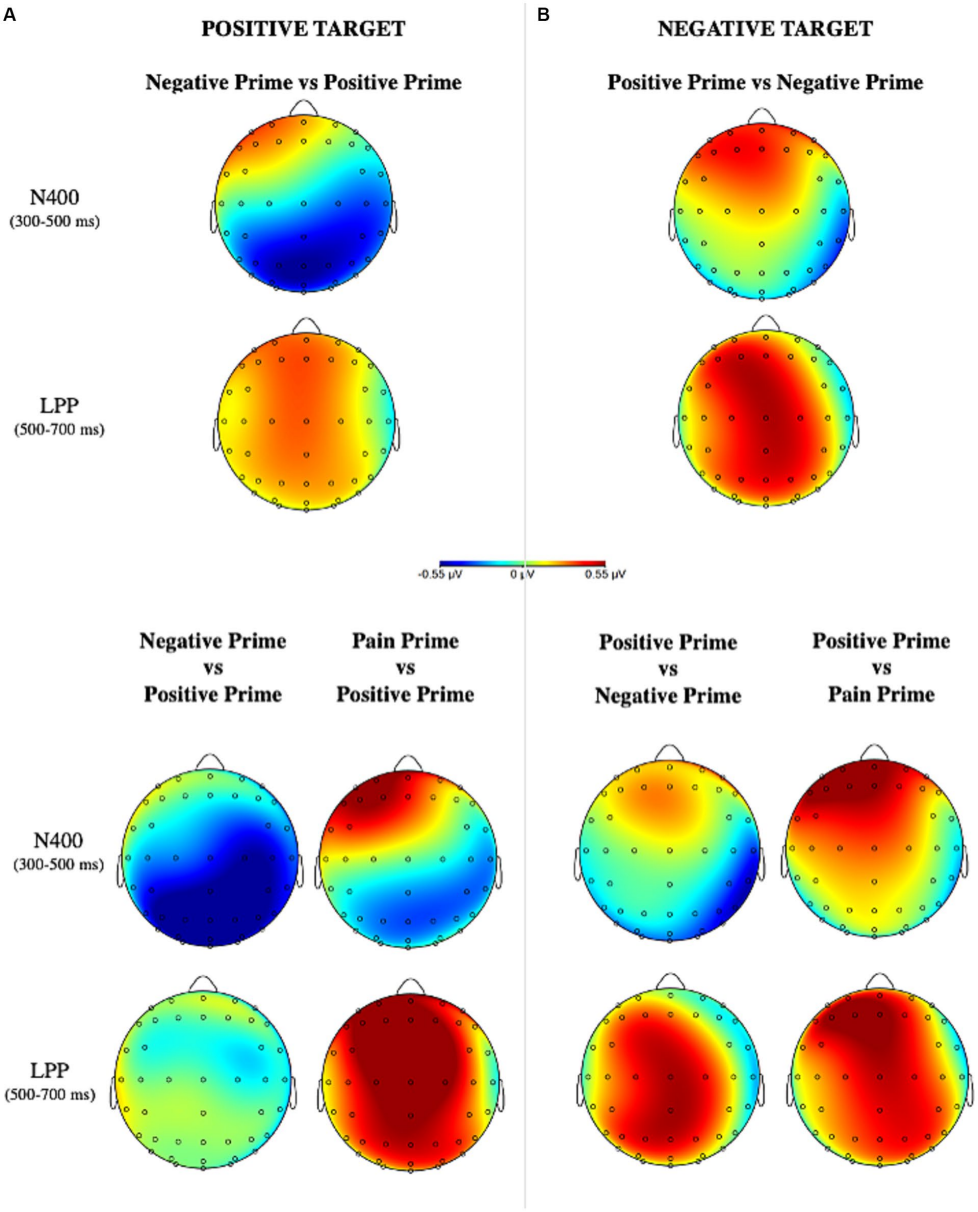
Topographical scalp distribution for positive and negative targets for the valence manipulation conditions **(A)** and for the semantic manipulation condition **(B)** in the two critical time windows, created by subtracting incongruent conditions and congruent conditions.

Grand-averaged ERPs elicited by the different experimental conditions are represented in Figure 4 and their topographical maps in Figure 5.

#### 3.2.1. N400

To investigate the role of prime valence, we performed repeated-measures 2x2x3x3 ANOVAs on ERPs amplitudes which showed a marginally significant main effect of prime valence [*F*(1,33) = 3.63; *p* = 0.066; η_p_^2^ = 0.1] so that the negative prime elicited larger negative waveforms (μV = 1.05, *SE* = 0.32) rather than the positive one (μV = 1.21, *SE* = 0.32). The analysis also showed the following significant interactions: target valence x longitude [*F*(1.41, 46.66) = 7.22; *p* = 0.005; ηp^2^ = 0.18]; latitude x prime valence x target valence [*F*(1.82, 60.16) = 4.32; *p* = 0.02; η_p_^2^ = 0.12]; longitude x prime valence x target valence [*F*(1.33, 43.8) = 11.99; *p* ≤ 0.001; η_p_^2^ = 0.27]. To further explore these interactions, the ERPs amplitudes of positive target and negative target were analyzed separately. The 2 × 3 × 3 ANOVA on the positive targets showed a significant longitude x prime valence interaction [*F*(1.28, 42.34) = 10.37; *p* = 0.001; η_p_^2^ = 0.24]. *Post-hoc* analyses revealed a larger negative waveform when the positive target is preceded by a negative prime (μV = 0.99, *SE* = 0.43) rather than a positive prime (μV = 1.46, *SE* = 0.43) at posterior positions.

Considering the negative target, the 2 × 3 × 3 ANOVA on the negative targets showed the subsequent significant interactions: latitude x prime valence [*F*(1.87, 61.65) = 6.02; *p* = 0.005; η_p_^2^ = 0.15]; longitude x prime valence [*F*(1.41, 46.59) = 4.09; *p* = 0.036; η_p_^2^ = 0.11]. No effects in the *post-hoc* analyses resulted significant.

To investigate the role of the prime semantics, a further 3 × 2 × 3 × 3 ANOVA showed the following significant interactions: prime semantics x target valence [*F*(1.67, 55.06) = 5.38; *p* = 0.011; η_p_^2^ = 0.14], longitude x target valence [*F*(1.45, 47.81) = 13.86; *p* ≤ 0.001; ηp^2^ = 0.3], longitude x prime semantics x target valence [*F*(2.5, 82.6) = 4.52; *p* = 0.009; η_p_^2^ = 0.12]; and a marginally significant latitude × longitude x prime semantics × target valence [*F*(4.7, 155.45) = 2.1; *p* = 0.074; η_p_^2^ = 0.06]. To further explore these interactions, the ERPs amplitudes of positive and negative targets were analyzed separately. The 3 × 3 × 3 ANOVA on the positive targets showed a significant main effect of prime semantics [*F*(1.92, 63.35) = 5.48; *p* = 0.007; η_p_^2^ = 0.14] so that the positive target elicited larger negative waveforms when preceded by a negative prime (μV = 0.91, *SE* = 0.35) rather than a pain one (μV = 1.31, *SE* = 0.35), and when it was preceded by a negative prime rather than a positive one (μV = 1.29, *SE* = 0.35). The analysis also showed a significant longitude x prime semantics interaction [*F*(2.44, 80.64) = 3.71; *p* = 0.021; η_p_^2^ = 0.1]. *Post-hoc* analyses revealed a larger negative waveform when the positive target is preceded by a negative prime (μV = 0.8, *SE* = 0.43) rather than a positive one (μV = 1.46, *SE* = 0.43) at posterior positions.

The 3x3x3 ANOVA on the negative targets showed a marginally significant latitude x longitude x prime semantics interaction [*F*(5.62,185.55) = 2.17; *p* = 0.052; η_p_^2^ = 0.06]. No significant *post-hoc* analyses resulted significant.

#### 3.2.2. LPP

To investigate the role of prime valence, we performed repeated-measures 2x2x3x3 ANOVA on ERPs amplitudes which showed the following significant interactions: prime valence x target valence [*F*(1, 33) = 7.38; *p* = 0.010; η_p_^2^ = 0.18], longitude x target valence [*F*(1.44, 47.54) = 11.95; *p* ≤ 0.010; ηp^2^ = 0.27], and a marginally significant latitude x prime valence x target valence interaction [*F*(1.7, 56.08) = 3.20; *p* = 0.056; η_p_^2^ = 0.09]. To further explore these interactions, the ERPs amplitudes of positive target and negative target were analyzed separately. The 2 × 3 × 3 ANOVA on the positive targets showed a significant main effect of the prime valence [F(1, 33) = 4.21; *p* = 0.048; η_p_^2^ = 0.11] in which the positive target elicited larger positive waveforms when preceded by a negative prime (μV = 2.33, *SE* = 0.46) rather than a positive prime (μV = 2.07, *SE* = 0.46). The 2x3x3 ANOVA on the negative targets showed a significant main effect of prime valence [F(1, 33) = 6.13; *p* = 0.019; η_p_^2^ = 0.16] in which the negative target elicited larger positive waveforms when preceded by a positive prime (μV = 2.58, *SE* = 0.41) rather than a negative one (μV = 2.23, *SE* = 0.41). In addition, the analysis also showed a significant latitude x prime valence interaction [*F*(1.82, 60.11) = 4.83; *p* = 0.014; η_p_^2^ = 0.13]. *Post-hoc* analyses revealed larger positive waveforms when the negative target is preceded by a positive prime (μV = 2.92, *SE* = 0.43) rather than a negative (μV = 2.44, *SE* = 0.43) one at midline positions.

Thereafter, to investigate the role of the prime semantics, a further 3x2x3x3 ANOVA was performed which showed the following significant interactions: prime semantics x target valence [*F*(1.83, 60.22) = 8.40; *p* ≤ 0.001; η_p_^2^ = 0.2], longitude x target valence [*F*(1.44, 47.44) = 11.44; *p* ≤ 0.001; ηp^2^ = 0.26], and a marginally significant latitude × longitude × prime semantics × target valence interaction [*F*(4.78, 157.45) = 2.21; *p* = 0.059; η_p_^2^ = 0.06]. To further explore these interactions, the ERPs amplitudes of positive target and negative target were analyzed separately. The 2x3x3 ANOVA on the positive targets showed a significant main effect of prime valence [*F*(1.95, 64.45) = 8.99; *p* ≤ 0.001; η_p_^2^ = 0.21] with larger positive waveforms when preceded by a pain prime (μV = 2.6, *SE* = 0.46) rather than a negative one (μV = 2.06, *SE* = 0.46) and a positive one (μV = 2.06, *SE* = 0.46). The 2 × 3 × 3 ANOVA on the negative targets showed a marginally significant main effect of prime semantics [*F*(1.98, 65.48) = 3.08; *p* = 0.053; η_p_^2^ = 0.085] so that the negative target elicited larger positive waveforms when preceded by a positive prime (μV = 258, *SE* = 0.41) rather than a pain prime (μV = 2.18, *SE* = 0.41). Moreover, the analysis also showed a significant latitude × prime semantics interaction [*F*(3.59, 118.41) = 2.83; *p* = 0.032; η_p_^2^ = 0.08] and only a marginally significant latitude × longitude × prime semantics interaction [*F*(5.17, 170.69) = 2.00; *p* = 0.079; ηp^2^ = 0.06]. No effects in the *post-hoc* analyses resulted significant.

### 3.3. Correlations

To test the influence of individual differences in pain processing on the accuracy and RT effects, we performed the correlation between the scores in the questionnaires’ subscales and the behavioral effects both for prime valence and prime semantics. In the analysis on prime valence, the correlation between the questionnaire’s subscales and the difference in accuracy scores Δ between congruent and-incongruent conditions showed that both the priming effects associated with the negative target and the positive target were positively correlated to the subscale Magnification of the PCS questionnaire (respectively Spearman’s rho = 0.42, *p* = 0.00137; Spearman’s rho = 0.350, *p* = 0.042).

In the analysis on prime semantics, the correlation analysis on accuracy scores showed a negative correlation between the priming effect associated with the negative target (pain prime-negative target vs. positive prime-negative target) and the subscales of the Empathic Concern (Spearman’s rho = −0.33, *p* = 0.027) and the Perspective Taking (Spearman’s rho = −0.37, *p* = 0.03115) of the IRI questionnaire. An additional negative correlation was detected between the priming effect associated with the negative target (negative prime-negative target vs. positive prime-negative target) and the Magnification subscales of the PCS questionnaire (Spearman’s rho = −0.38, *p* = 0.02914). The correlational analyses on RTs did not show any significant results.

As well we analyzed the individual differences in pain processing on the ERP effect. In the analysis on prime valence for both the ERP components, the correlation analysis between questionnaires’ subscales and the difference in mean amplitudes for all electrode sites Δ between congruent and-incongruent conditions did not show any significant results. In the analysis on the prime semantics, the correlation analyses on N400 mean amplitudes showed that the priming effects associated with the positive target (positive prime-positive target vs. negative prime-positive target) correlated with both the BIS scale (Spearman’s rho = 0.43, *p* = 0.012), the Personal Distress subscale of the IRI questionnaire (Spearman’s rho = 0.40, *p* = 0.019), and the Rumination subscale of the PCS questionnaire (Spearman’s rho = 0.354, *p* = 0.04). The correlation analyses on LPP mean amplitudes showed that the priming effects associated with the negative target (pain prime-negative target vs. negative prime-negative target) correlated with both the Fantasy subscale of the IRI questionnaire (Spearman’s rho = 0.35, *p* = 0.04), and the Rumination subscale of the PCS questionnaire (Spearman’s rho = 0.38, *p* = 0.029).

## 4. Discussion

In the present experiment, we explored the time course of the implicit processing of pain words, particularly whether the processing of a stimulus semantically associated to pain can help the individual to respond to an upcoming negative information in the environment. To our knowledge, our study represents the first to adopt the well-known affective priming paradigm combined with EEG recordings to investigate the neural correlates of the elaboration of pain words.

At the behavioral level, results confirmed what we have already found in our previous study using the same paradigm (Gilioli et al., 2023). They showed an affective priming, that is the participant responded faster to the target when this was preceded by a prime of the same valence. The affective priming effect for positive congruent conditions confirmed the effect already described in the literature (Aguado et al., 2013; Contreras-Huerta et al., 2013; Gibbons et al., 2014), whereas the affective priming for negative congruent conditions supports the hypothesis according to which a negative prime may facilitate the response to a negative target (Meagher et al., 2001). A subsequent analysis considering separately negative prime and pain prime revealed that the affective priming effect described above emerged only in the condition in which the negative target was preceded by a pain prime but not by a negative prime. Thus, the semantics of pain embodied in the prime would therefore appear to have facilitated the processing of the negative target.

ERPs findings allowed a deeper understanding of the underlying mechanism of this effect. At an earlier stage of stimulus processing, our data showed a significant effect on the N400 for the positive target with larger negativity when it is preceded by a negative prime (affective incongruency) rather than a positive one (affective congruency), in accordance to what had already been found in the literature (Zhang et al., 2006, 2010; Eder et al., 2012). Against our expectation, no effect has been detected for the negative target. Once the semantic of pain was entered in the analyses, it is interesting to see how the N400 component had larger amplitude when the positive target was preceded by a negative prime at posterior scalp positions rather than a positive prime or a pain prime. Again, no effect was found for the negative target. At first glance, at an early time window (300–500 ms) the semantic of pain is not playing any role in guiding the processing of upcoming information. Indeed, on the positive target, the N400 which primarily reads the semantic incongruency between stimuli is mainly elicited by a negative prime and not by a pain prime. Moreover, this effect was detected at the posterior scalp locations (Figures 4,5) restating previous findings of affective priming on words stimuli (Kissler et al., 2009; Zhang et al., 2010). This may also have depended by the visual modality of the stimuli (Zhang et al., 2006; Kutas and Federmeier, 2011; Eder et al., 2012) and, in particular, by the involvement of posterior areas during the perceptual analysis of word strings (Ponz et al., 2014).

At a later stage of stimulus elaboration, the affective incongruent conditions elicited a greater positivity on the LPP component for both positive and negative targets in agreement with previous studies on affective priming. Additionally, considering the semantic of pain it emerged that for the positive target, this effect was entirely driven by the pain prime: in fact, a positive waveform was elicited when a positive target was preceded by a pain prime but not by a negative prime. As well, a greater LPP was detected when the negative target was preceded by a positive prime rather than a pain prime. It is possible to speculate that the semantics of pain needs the allocation of more attentional resources to be elaborated, thus, influencing the subsequent response to a target information. It is well-known that a greater LPP is usually elicited by the inconsistency of valence due to the increased attentional resources (Kissler et al., 2009; Zhang et al., 2010). This component is indeed involved in tasks of attention, evaluation, and memory encoding (Kissler et al., 2009).

Considering that our study was the first to investigate the neural correlates of pain words using this paradigm, our findings need to be further interpreted. At an earlier stage (N400) of processing, the majority of ERPs studies on affective priming usually reported larger negativity in affectively incongruent conditions highlighting the sensitivity of the N400 to the semantic relatedness and congruency between the prime and the target (Zhang et al., 2006, 2010; Steinbeis and Koelsch, 2011; Eder et al., 2012). This might be read in the context of the spreading activation within the semantic network (Fazio et al., 1986; Murphy and Zajonc, 1993). Nevertheless, there is also additional evidence showing no effects (Herring et al., 2011; Kissler and Koessler, 2011) or even a reverse N400 effect (Paulmann and Pell, 2010; Aguado et al., 2013; Wang and Zhang, 2016) with a larger negativity for affectively congruent trials.

According to the literature, the N400 has also a role in the processing of integrating a target stimulus into the preceding context given by the prime. Embedding the target into the context may entangle two levels of affective evaluation: the first regards the elaboration of the valence, and the second regards the elaboration of the semantics of the stimulus (Aguado et al., 2013). The result of this dual evaluation turns out to differ for positive and negative emotional stimuli. For instance, a study by Aguado et al. (2013) showed how a positive facial expression may be representative of several positive emotions so that it can be easily embedded within a large variety of positive target words. On the contrary, the integration of a target into a negative context (e.g., anger) requires the individual to distinguish among a broad range of emotional contents activated by negative valence stimuli. The high demands of this task may require the individual more time to be able to discriminate among the negative affective domain (Aguado et al., 2013). This may account for the inconsistency of results found in the literature regarding the affective priming for negative stimuli: the heterogeneity of the semantics embraced in the negative valence could have limited the emergence of the affective priming for the negative target (Rossell and Nobre, 2004). Nevertheless, it is worth pointing out that the semantics of pain needs more time to be elaborated on due to the necessity of additional attentional resources as a result of the specificity of the affective content that characterized it (Kissler et al., 2009; Zhang et al., 2010). This reaffirms the great sensitivity of the N400 discriminating the semantic content of the stimuli rather than just their valence.

However, at a later stage (LPP) of stimulus processing, the cognitive system is prepared to elaborate the evaluative properties of the stimuli generating peculiar effects according to the affective value of the stimulus (Herring et al., 2011). At this time, both positive and negative stimuli showed greater positive waveforms in the affectively incongruent conditions. Importantly, it is worth highlighting that these effects were entirely guided by the semantic of pain embedded in the prime: indeed, as soon as we considered it in the analyses, the LPP component resulted larger only when the positive target was preceded by a pain prime and not by a negative one. As well it is larger when the negative target was preceded by a positive prime rather than a pain one, and no significant effect was detected instead when the negative target was preceded by a positive prime rather than a negative one. These findings confirmed the involvement of the LPP during the processing of emotionally salient stimuli showing its role in generating a specific response to each type of emotion, in particular, it is clear how the effect on this component is due to the semantics of pain. Thus, if on one hand individuals are engaged in resolving the conflict between the semantics and the valence of a stimulus in the time window between 300 and 500 ms, then in the interval between 500 and 700 ms they are engaged in producing affective responses peculiar to each emotional content.

Besides positive and negative stimuli are differently processed in the brain, potentially due to the involvement of different brain areas (Comesaña et al., 2013), the relevance of considering the extreme heterogeneity of semantic contents among negative stimuli has largely been discussed in other previous studies on affective priming (Rossell and Nobre, 2004; Aguado et al., 2018; Gilioli et al., 2023). Indeed, pairs of words belonging to “fear” category generate a modest priming effect on negative targets (Rossell and Nobre, 2004). Conversely, pairs of words belonging to the “sadness” category produced an inhibiting effect on the processing of pain targets (Song et al., 2019). It follows that affective categories within negative valence should be considered separately, which is why results are so inconsistent (Paulmann and Pell, 2010; Herring et al., 2011; Eder et al., 2012; Aguado et al., 2013).

Although the affective priming research has been mainly focused on the role of the prime in influencing the response to the target, it has been stated that also the target can intervene in this effect (Chan et al., 2006). Results from the study showed an affective priming effect for low frequency target words and a reverse priming effect for high frequency target words. Despite in our study the familiarity of the targets, a good estimate of the frequency (Leroy and Kauchak, 2014), did not significantly covariate with the affective priming effect, the arousal and the length of the target words did significantly covariate. Nevertheless, they have not interfered with the interaction between the prime and the target which was the main focus of our analysis.

Eventually, a parameter that may have played a role in these results is the stimulus onset asynchrony (SOA) which is the interval between the prime and the target onset. Indeed, a previous study by Paulmann and Pell (2010) found a greater N400 for affective incongruent trials at 400 ms SOA and a reverse N400 effect for congruent trials at 200 ms SOA. It is reasonable to think that in our study using a SOA of 300 ms may have contributed to generating this complex pattern of results. Future studies should take this variable into account.

In addition, another limitation of our experiment was the recruitment of a sample composed only by females. Indeed, other studies reported gender differences using this particular paradigm, with stronger effects in female than male participants (Hermans et al., 1998; Schirmer et al., 2005). Moreover, gender differences have been extensively covered by studies on pain processing (Rhudy and Williams, 2005). Much research has shown that females reported more intense reactions to pain stimuli (Rhudy and Williams, 2005), and even a different perception of risk than males (Charness and Gneezy, 2012). Based on these differences and our previous study using the same paradigm (Gilioli et al., 2023), we initially preferred to focus on females to maximize a possible effect, but for generalizability of the results, there is the need to extend the study to males.

To sum up, the ERPs components analysis gave an interesting insight on the time course of the implicit processing of pain. It turned out that the time window between 300 and 500 ms is crucial to studying the interaction between the semantics and the valence of a stimulus. Even more, it restated the importance of considering the semantics of negative stimuli. In fact, at this time, the semantics of pain of the prime required the allocation of more cognitive resources to be elaborated among the heterogeneous groups of emotional contents of negative stimuli. The more the stimulus processing progresses in time, the more the cognitive system is able to recognize the adaptive value of the pain content pre-activating the individual to respond as quickly as possible to un upcoming negative information, as behavioral findings showed. Indeed, the time window between 500 and 700 ms turns out to be extremely sensitive to generate specific responses to each affective and emotional information. As already stated by Herring et al. (2011), we can speculate that the N400 is more sensitive to the evaluation of the semantics of stimuli and the LPP to their affective evaluation.

Ultimately, it is possible that the double nature of pain itself may have contributed to generating this complex pattern of results. According to the motivational priming theory (Lang, 1995; Davidson and Irwin, 1999; LeDoux, 2000), pain has specific properties, and its elaboration may promote the survival of the individual both by facilitating the individual to respond faster to aversive signals, both by supporting approach responses to others’ pain.

However, individual differences in pain processing may account for the effect as showed by correlation analyses. In particular, on behavioral results, the correlation of the affective priming and the Magnification subscale of the PCS suggested that the individual tendency to amplify the severity of negative stimuli may have influenced the response to the target. In particular, the correlation between the negative priming associated to pain prime and the Perspective Taking subscale of IRI proposes a relation with the capacity of feeling compassion for others. On ERP results, the N400 elicited by the negative prime on the positive target might have been influenced by the tendency of an individual to respond to threat signals (BIS scale) and to feel personal distress (IRI Personal Distress subscale).

On the other side, the LPP on the negative target correlated with the tendency of the individual to get involved in vivid and imaginative fantasies (IRI Fantasy subscale). Both the N400 and the LPP seemed to be impacted by the tendency of an individual to ruminate about negative thought (Rumination subscale of PCS). The individual influences on the affective priming related to pain prime especially on the LPP restated that the role of the component in the elaboration of emotionally salient stimuli can be top-down modulated by the subjective interpretation of the stimuli (Hartigan and Richards, 2016).

In the present study, the category of negative, pain-unrelated words included words belonging to different semantic contents. In future studies, it would be of interest to compare pain-related words to other defined semantic categories, like other negative emotions, as they may represent more appropriate comparisons. However, not all words may be unambiguously categorized into a discrete emotion or a specific semantic content, raising concerns about statistical power (Witherell et al., 2012; Kveraga et al., 2015). Defining an appropriate paradigm, experimental design, normative data, and statistical analysis are crucial aspects that researchers should carefully consider avoiding this potential problem. For instance, a paradigm that includes contextual information to aid the disambiguation of semantic content may be useful for better accuracy. Collecting normative data may also help categorize the semantic content of each stimulus and prevent extraneous sources of variation.

In conclusion, although some ERPs results do not survive correction for multiple comparisons and we are aware that cautious interpretations are needed, this study represents the first data on the topic. It provides a small contribution to studying the process of sensorimotor resonance between oneself and others, also called empathy, that allows understanding the other through the vicarious sharing of their emotional experiences and beliefs (Betti and Aglioti, 2016).

## Data availability statement

The original contributions presented in the study are included in the article/supplementary material, further inquiries can be directed to the corresponding authors.

## Ethics statement

The studies involving humans were approved by Ethical Committee of the University of Modena and Reggio Emilia. The studies were conducted in accordance with the local legislation and institutional requirements. The participants provided their written informed consent to participate in this study.

## Author contributions

AG, EB, LS, and FP conceptualized the research study and design, interpreted the results and wrote the manuscript. FP and AG programmed the experiment and performed the analysis. AG and EB coordinated participants’ recruitment and data collection. All authors contributed to the article and approved the submitted version.

## Funding

This research was funded by the FAR2022INTERM_O_UNIM grant from the University of Modena and Reggio Emilia (https://www.unimore.it).

## Conflict of interest

The authors declare that the research was conducted in the absence of any commercial or financial relationships that could be construed as a potential conflict of interest.

## Publisher’s note

All claims expressed in this article are solely those of the authors and do not necessarily represent those of their affiliated organizations, or those of the publisher, the editors and the reviewers. Any product that may be evaluated in this article, or claim that may be made by its manufacturer, is not guaranteed or endorsed by the publisher.

## Footnotes

^1^ www.aipass.org/node/11560
