# Supplementary material for: Electrophysiological correlates of semantic pain processing in the affective priming
Source: Front Psychol. 2023 Sep 8;14:1201581. doi: 10.3389/fpsyg.2023.1201581 (PMC10516560; doi:10.3389/fpsyg.2023.1201581)
Supplement: Supplementary file 1 [file Table_1.pdf]

Supplementary Table 1. List of Italian words included in the stimulus sets. Words are divided into positive words, negative words not associated to pain, and negative words associated to pain and the English translation is reported.

**Positive words**		**Negative words not associated to pain**		**Negative words associated to pain**	
**Italian**	**English**	**Italian**	**English**	**Italian**	**English**
Agilità	Agility	Abbandonato	Abandoned	Aggressione	Aggression
Ambizione	Ambition	Alcolista	Alcoholic	Amputazione	Amputation
Ammirato	Admired	Angosciato	Anguished	Atroce	Atrocious
Amore	Love	Annoiato	Bored	Cancro	Cancer
Angelo	Angel	Ansioso	Anxious	Dilaniante	Excruciating
Appetitoso	Luscious	Arrabbiato	Angry	Dolore	Pain
Arte	Art	Assassino	Killer	Ferita	Wound
Atletica	Athletics	Avidità	Greed	Fitta	Stabbing pain
Attraente	Handsome	Avversione	Hatred	Frattura	Fracture
Aurora	Dawn	Bastardo	Bastard	Frustata	Lash
Avventura	Adventure	Bestemmia	Blasphemy	Incurabile	Incurable
Beatitudine	Bliss	Bollente	Hot	Infarto	Heart attack
Bebè	Baby	Bomba	Bomb	Infiammazione	Inflammation
Bellezza	Beauty	Carcere	Jail	Lacerante	Lacerating
Bello	Beautiful	Cattivo	Evil	Lacerazione	Laceration
Capace	Capable	Collera	Anger	Lancinante	Stabbing
Cascata	Waterfall	Colpevole	Guilty	Lesione	Lesion
Cervello	Brain	Corrotto	Corrupted	Male	Pain
Charme	Charm	Criminale	Criminal	Mutilazione	Mutilation
Ciclamino	Cyclamen	Crisi	Crisis	Piaga	Plague
Comfort	Comfort	Debito	Debt	Scorticamento	Flaying
Conoscenza	Knowledge	Demonio	Demon	Sevizia	Torture
Coraggioso	Brave	Disastro	Disaster	Sofferto	Suffered
Cortese	Kind	Discarica	Dump	Supplizio	Torment
Curioso	Curious	Disgustato	Disgusted	Tendinite	Tendinitis
Delizia	Delight	Distruzione	Destruction	Torturante	Torturing
Desiderio	Wish	Drogato	Drug addicted	Trafiggente	Piercing
Diamante	Diamond	Egoista	Selfish	Trafittivo	Excruciating
Dollaro	Dollar	Feccia	Scum	Trauma	Trauma
Dono	Present	Fetore	Stench	Ulcera	Ulcer
Eccellenza	Excellence	Frustrato	Frustrated	Ulcerativo	Ulcerative
Eccezionale	Outstanding	Ghigliottina	Guillotine	Ustione	Burn
Eccitato	Aroused	Grasso	Fat		
Eccitazione	Excitement	Guerra	War		
Elegante	Elegant	Ignoranza	Ignorance		
Erotico	Erotic	Immaturo	Immature		
Estasi	Ecstasy	Immondizia	Dirty		
Esultante	Elated	Impaurito	Scared		
Fantasia	Fantasy	Impotente	Impotent		
Favore	Favor	Incidente	Accident		
Ferie	Vacation	Incubo	Nightmare		
Festivo	Festive	Infedele	Unfaithful		
Fiducioso	Confident	Inganno	Deceit		
Forte	Strong	Inondazione	Flood		
Fortunato	Lucky	Insicuro	Insecure		
Fratello	Brother	Inutile	Useless		
Giocattolo	Toy	Maleducato	Rude		
Gioioso	Joyful	Malvagio	Wicked		
Gioviale	Jolly	Maniaco	Maniac		
Giustizia	Justice	Marcio	Rotten		
Gloria	Glory	Minaccia	Threat		
Godimento	Enjoyment	Miseria	Misery		
Grato	Grateful	Muffa	Mold		
Gusto	Taste	Nervoso	Nervous		
Idea	Idea	Obesità	Obesity		
Idolo	Idol	Odio	Hate		
Incentivo	Incentive	Odioso	Obnoxious		
Interesse	Interest	Omicida	Murderer		
Ispirato	Inspired	Orrore	Horror		
Leale	Loyal	Ostile	Hostile		
Liberazione	Liberty	Penalità	Penalty		
Libertà	Freedom	Perdente	Loser		
Luminoso	Bright	Pericolo	Danger		
Lusso	Luxury	Pessimismo	Gloom		

Positive words		Negative words not associated to pain		Negative words associated to pain	
Magico	Magical	Povertà	Poverty		
Magnifico	Terrific	Precipizio	Cliff		
Mente	Mind	Prigione	Prison		
Meraviglia	Wonder	Putrido	Putrid		
Milionario	Millionaire	Puzza	Stink		
Miracolo	Miracle	Rapimento	Abduction		
Mondo	World	Ratto	Rat		
Natura	Nature	Respinto	Rejected		
Neonato	Infant	Ricatto	Blackmail		
Neve	Snow	Sanguinoso	Bloody		
Oceano	Ocean	Schiavo	Slave		
Onore	Honor	Sconfitto	Defeated		
Orgasmo	Orgasm	Scoraggiato	Discouraged		
Oro	Gold	Sepoltura	Burial		
Ottimismo	Optimism	Sgradevole	Nasty		
Paradiso	Paradise	Sleale	Disloyal		
Passione	Passion	Solitudine	Loneliness		
Perspicacia	Wit	Spaventato	Scared		
Possibilità	Chance	Spazzatura	Garbage		
Prestigio	Prestige	Sporcizia	Filth		
Progresso	Progress	Sporco	Dirt		
Promozione	Promotion	Sudiciume	Grime		
Protetto	Protected	Terrorista	Terrorist		
Radioso	Radiant	Terrorizzato	Terrified		
Regalo	Gift	Traditore	Traitor		
Ricchezze	Riches	Tragedia	Tragedy		
Ricco	Wealthy	Truffa	Fraud		
Ricompensa	Reward	Vandalo	Vandal		
Riconoscente	Thankful	Vigliacco	Louse		
Rinfresco	Refreshment	Vittima	Victim		
Rispetto	Respect	Zanzara	Mosquito		
Romantico	Romantic				
Saggio	Wise				
Sesso	Sex				
Seta	Silk				
Sexy	Sexy				
Sicuro	Safe				
Soddisfatto	Satisfied				
Soleggiato	Sunlight				
Sorpreso	Surprised				
Sorriso	Grin				
Spensierato	Carefree				
Speranza	Hope				
Speranzoso	Hopeful				
Sposa	Bride				
Studioso	Scholar				
Successo	Success				
Talento	Talent				
Tesoro	Treasure				
Trionfante	Triumphant				
Trionfo	Triumph				
Trofeo	Trophy				
Umile	Humble				
Utile	Useful				
Vantaggio	Advantage				
Vigoroso	Vigorous				
Villaggio	Village				
Villetta	Cottage				
Vincitore	Champion				
Virtù	Virtue				
Vita	Life				
Vittoria	Victory				
Vivace	Lively				
Vivo	Alive				
